# The effects of supplementation of *Bacillus subtilis* and *Bacillus coagulans* spores on the intestinal microflora and growth performance in rat

**Published:** 2019-06

**Authors:** Somaye Mazkour, Seyed Shahram Shekarforoush, Sara Basiri

**Affiliations:** Department of Food Hygiene and Public Health, School of Veterinary Medicine, Shiraz University, Shiraz, Iran

**Keywords:** *Bacillus subtilis*, *Bacillus coagulans*, Intestinal microflora, Feed conversion ratio

## Abstract

**Background and Objectives:**

The intestinal microflora has an important role in the health status. Since probiotics can balance the intestinal microflora, they have a lot of health beneficial effects. So the appropriate selection of probiotics can cause health-promoting effects. In this study, the combined effects of *Bacillus subtilis* and *Bacillus coagulans* on the intestinal microflora and growth performance in rats were investigated.

**Materials and Methods:**

80 male rats were divided into the treatment (receiving 5×10^7^ spores/ml of *B. subtilis* and 5×10^7^ spores/ml of *B. coagulans* for three weeks in daily water) and control (tap water without probiotics) groups. The total aerobic and anaerobic microorganisms, lactic acid bacteria (LAB), coliforms and spores were weekly counted in the fecal samples. Additionally, the water and feed consumption, the weight gain and Feed Conversion Ratio (FCR) were calculated for each week.

**Results:**

The probiotics significantly increased the total aerobic, LAB and spore counts and caused significant reduction in the anaerobe and coliform counts. Following three weeks of probiotic administration, the number of anaerobic bacteria, and coliforms were reduced by up to 0.7 and 1.18 log and the number of aerobic bacteria, LAB and spores were increased by 1.29, 1.15 and 7.2 log respectively. Also, the results showed the feed consumption reduction, weight gain and FCR enhancement in the probiotic group (*p* < 0.05).

**Conclusion:**

Supplementation the spores of *B. subtilis* and *B. coagulans* improved the growth performance and was beneficial to the intestinal microbiota in rats.

## INTRODUCTION

The intestinal microflora and their productions are effective in the health (1). They make a primary barrier that protects the body from pathogens (2, 3), but various diseases disrupt the balance of the normal intestine microflora (3). Probiotics, mostly lactic acid bacteria such as lactobacilli and bifidobacteria reduce intestinal infectious diseases (4, 5). Probiotics are living microorganisms that at specific concentrations have beneficial effects on human health by improving the intestinal microbial balance (6, 7). They also prevent the colonization of pathogenic bacteria in the digestive tract, compete for nutrients and adhesion receptors, and stimulate host immunity (4, 6, 8, 9). Various species of *Bacillus, Bifidobacterium, Enterococcus, Lactobacillus, Lactococcus, Streptococcus* as well as some yeasts such as *Saccharomyces cerevisiae* have been used as probiotics. These are very useful even in comparison with antibiotics (10–13). Spores of *Bacillus spp.* commercially used as probiotics, and unlike *Lactobacillus spp.*, they are dormant (13). Spore former probiotics, as compared to non-spore formers, have some advantages such as resistance to gastric acid. Accordingly, they will be able to pass through the stomach, and in a concentration similar to the initial concentration will enter the intestine. But active probiotics are susceptible to gastric acid and bile salts, and their numbers decrease in the gastrointestinal tract (14–17). Therefore, spore species are of great importance due to the high vitality of spores (18). Some researchers have evaluated the effects of spore forming and non-spore forming probiotics on intestinal microbiota. Most of them have shown that probiotics enhance the beneficial bacteria such as lactic acid bacteria and reduce the harmful ones such as coliforms in the intestine by more than 1 log cycle (17, 19). The *Bacillus* genus has potentially useful metabolic substances and enzymatic ability. As endospore formers, they are also resistant microorganisms and survive in a variety of food products compared to the more typical probiotic species (20). The most comprehensively investigated species of spore-forming *Bacillus* include *B. licheniformis, B. clausii, B. coagulans, B. cereus* and *B. subtilis* (21). Of hundreds of known *Bacillus spp.*, only *B. coagulans* and *B. subtilis* var. *natto* have generally been accepted as admissible probiotics for human consumption. Some of the beneficial effects of these species are individually examined. *B. subtilis* var. *natto* is believed to stimulate the immune system, pose anti-cancer properties and produce vitamin K2 and *B. coagulans* was described as an aid for the absorption and utilization of proteins (22–24). Also, *B. subtilis* improved the feed conversion ratio (FCR) and average weight gain in the broiler chicken (25, 26). However, combination effects of *Bacillus spp.* spores have not been investigated enough before. Haldar and Gandhi examined the effect of oral administration of *B. coagulans* and *B. pumilus* on intestinal microflora (17). So, we conducted this study to evaluate the *in vivo* effects of spores of *Bacillus subtilis* and *Bacillus coagulans* in combination on the normal intestinal microflora and growth performance.

## MATERIALS AND METHODS

### Preparation of probiotic bacteria.

Spray dried dextrose powder containing 10^10^ spores per gram of *B. subtilis* (PRM102) and *B. coagulans* (PRM101) were donated by the Pardis Roshd Mehregan Company, Iran. To confirm the spore concentrations of the probiotics, one gram of each powder was dissolved in 100 ml tap water (10^8^ spores/ ml) and heated at 80°C for 15 min, to kill the vegetative cells, and surface plating after tenfold serial dilution was prepared on the Plate Count Agar (Merck, Germany). Fresh spore suspensions at the concentration of 5×10^7^ of each spores/ml was prepared daily in 200 ml tap water.

### Experimental design.

In this experimental study, 80 male Spargue-Dawley rats weightening 170–190 gr were procured from Department of Animal Lab, Shiraz University of Medical Sciences, Iran. They were housed in plastic cages and kept under 12-hour light/ dark condition, temperature of 20–25°C, humidity of 50–60%, and free access to food and water in order to adaptation to the new environment. The animals were fed with commercially standard pellet.

After an acclimatization period of 1 week, they were randomly divided into two groups (each group consists of 10 subgroups- 4 rats in each subgroup). Control group: Received water containing 1% dextrose for 3 weeks. Probiotic group: Receiving water containing 5×10^7^ spores/ml of *B. subtilis* and 5×10^7^ spore/ml of *B. coagulans* for 3 weeks.

### Growth and diet indexes.

The rats were weighted every week and water and feed consumption was measured every week for each cage to evaluate the effect of *B. subtilis* and *B. coagulans* in combination on growth performance. Finally the feed conversion ratio (FCR) was calculated for each subgroup weekly by using the following equation (27):

$$\begin{array}{l} \text{FCR}=\;\text{Average}\;\text{feed}\;\text{intake}\;\text{per}\;\text{subgroup}\;\text{per}\;\text{week}\\ \left(\text{g})\;/\;\text{Average}\;\text{weight}\;\text{gain}\;\text{per}\;\text{subgroup}\;\text{per}\;\text{week}(\text{g}\right) \end{array}$$

### Intestinal microflora analysis.

On days 0, 7, 14 and 21, a pooled fecal sample from the rats in each subgroup was taken. Just after collection, each subgroup’s fecal sample was weighed and homogenized in sterile phosphate buffer saline (PBS), and then, tenfold serial dilution was made in PBS. Aerobic and anaerobic microorganisms were cultured in Plate Count Agar (Merck, Germany). Lactic acid bacteria (LAB) were cultured in MRS (de Man, Rogosa, Sharpe) agar (Merck, Germany). Coliforms were cultured in VRBL (Violet Red Bile Lactose) agar (Merck, Germany). LAB and anaerobic bacteria were incubated at anaerobic condition. All plates were incubated at 37°C for 48 h. The microflora enumeration was expressed as CFU/gr of feces. In order to count bacterial spores in fecal samples, the homogenized samples in PBS were put into a water bath at 80°C for 10 min, and then cooled immediately to help the spore germination. Then the samples were serially diluted in PBS and cultured in Plate Count Agar (Merck, Germany). The plates were incubated at 37°C for 48 h. The spore enumeration was expressed as spores/gr. All the samples were cultured in duplicate (17, 19).

### Ethical approval.

The trial was permitted by the commission on animal ethics, Shiraz University, Shiraz, Iran (Ethical approved number: 1395/9234308).

### Statistical analysis.

The results were analyzed using analysis of variance and the statistical significance of differences between mean values was analyzed by Duncan’s multiple range tests. P-values less than 0.05 were considered statistically significant. Analysis was performed using the statistical Package for Social Sciences (SPSS) software (SPSS 16 for windows, SPSS Inc, Chicago, IL, USA).

## RESULTS

### Growth and diet indexes.

The rats were weighted every week and water and feed consumption was measured for each cage and the FCR for each cage was calculated. The results are shown in Table 1. Consumption of *B. subtilis* and *B. coagulans* caused a significant enhancement in the weight gain in rats after two weeks. At the 3^rd^ week it was 58.5 in control group versus 64.7 in probiotic group. The amount of feed intake decreased after one week of consumption. The difference between goups was about 14.5 gr for each rat per week. After three weeks of treatment, FCR in the probiotic group was significantly lower than the control group (*p* < 0.05). Although, the administration of *B. subtilis* and *B. coagulans* did not have a significant effect on the amount of water intake (*p* > 0.05), it was significantly reduced the food intake in the second and third weeks of the study (*p* < 0.05).

**Table 1.** The amount (mean±SD) of feed intake, weight gain, feed conversion ratio (FCR) and water consumption per subgroup (4 rats) in control and probiotic groups during three weeks of treatment.

**Factors**	**Weeks**	**Groups**	

		**Control**	**Probiotic**
Feed intake (gr/week)	1	534.8±33.4^a^	500.6±41.3^a^
	2	563.0±23.3^a^	518.4±29.0^b^
	3	593.4±45.1^a^	520.6±27.0^b^
Weight gain (gr/week)	1	46.2±4.0^a^	48.7±2.5^a^
	2	54.4±5.1^a^	54.5±3.7^a^
	3	58.5±3.7^a^	64.7±2.8^b^
FCR	1	11.7±1.4^a^	10.3±1.1^b^
	2	10.4±1.0^a^	9.5±0.7^a^
	3	10.2±1.0^a^	8.2±0.7^b^
Water consumption (ml/week)	1	1282.0±148.5^a^	1283.3±93.5^a^
	2	1302.0±176.7^a^	1277.8±106.4^a^
	3	1261.0±214.1^a^	1288.9±105.4^a^

Control: Received water for 3 weeks. Probiotic: Receiving water containing 5×10^7^ spores/ml of *B. subtilis* and 5×10^7^ spores/ml of *B. coagulans* for 3 weeks; The different letters indicate significant differences between groups (*p* < 0.05)

### Intestinal microflora analysis.

**Fig. 1.**
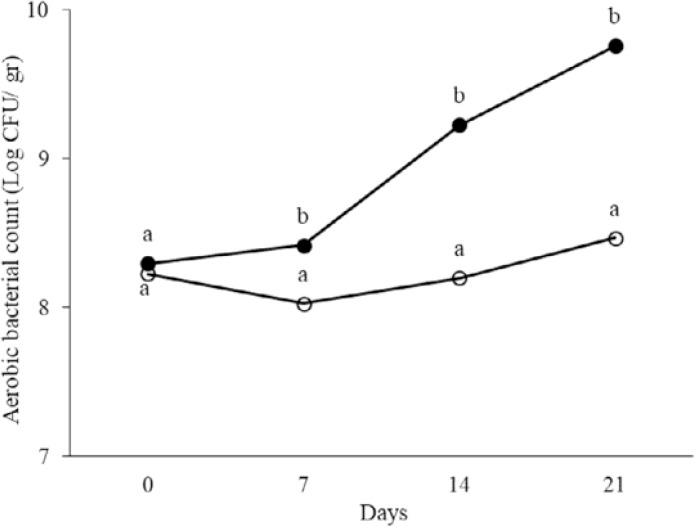
The logarithm of aerobic bacterial count in fecal samples of rat in control (○) and probiotic (●) groups. Different letters indicate significant differences between groups in each day (*p* < 0.05). Control: Received water for 3 weeks. Probiotic: Receiving water containing 5×10^7^ spores/ml of *B. subtilis* and 5×10^7^ spores/ml of *B. coagulans* for 3 weeks

**Fig. 2.**
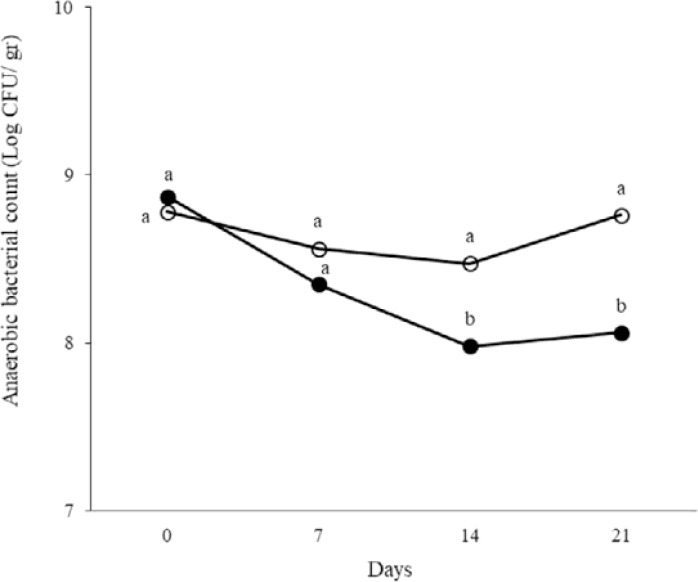
The logarithm of anaerobic bacterial count in fecal samples of rat in control (○) and probiotic (●) groups. Different letters indicate significant differences between groups in each day (*p* < 0.05). Control: Received water for 3 weeks. Probiotic: Receiving water containing 5×10^7^ spores/ml of *B. subtilis* and 5×10^7^ spores/ml of *B. coagulans* for 3 weeks

**Fig. 3.**
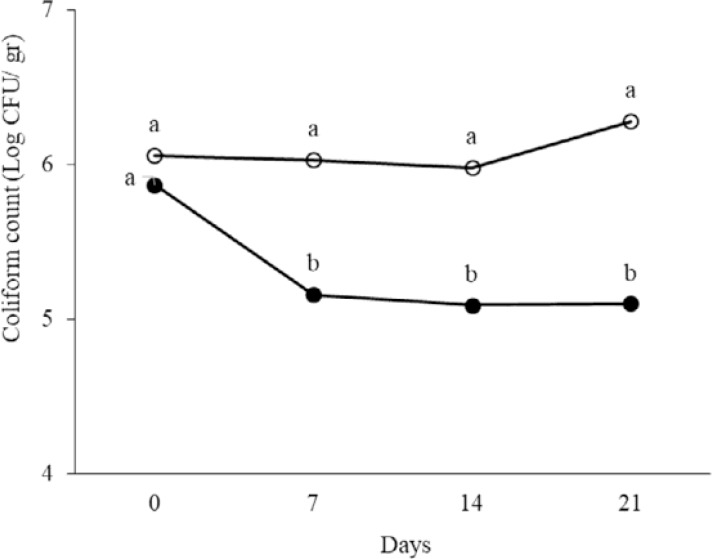
The logarithm of coliform count in fecal samples of rat in control control (○) and probiotic (●) groups. Different letters indicate significant differences between groups in each day (*p* < 0.05). Control: Received water for 3 weeks. Probiotic: Receiving water containing 5×10^7^ spores/ml of *B. subtilis* and 5×10^7^ spores/ml of *B. coagulans* for 3 weeks

**Fig. 4.**
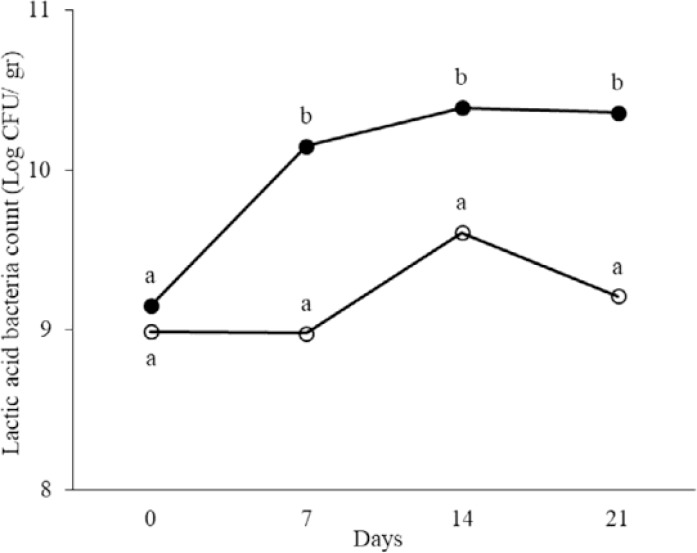
The logarithm of lactic acid bacteria count in fecal samples of rat in control (○) and probiotic (●) groups. Different letters indicate significant differences between groups in each day (*p* < 0.05). Control: Received water for 3 weeks. Probiotic: Receiving water containing 5×10^7^ spores/ml of *B. subtilis* and 5×10^7^ spores/ml of *B. coagulans* for 3 weeks

**Fig. 5.**
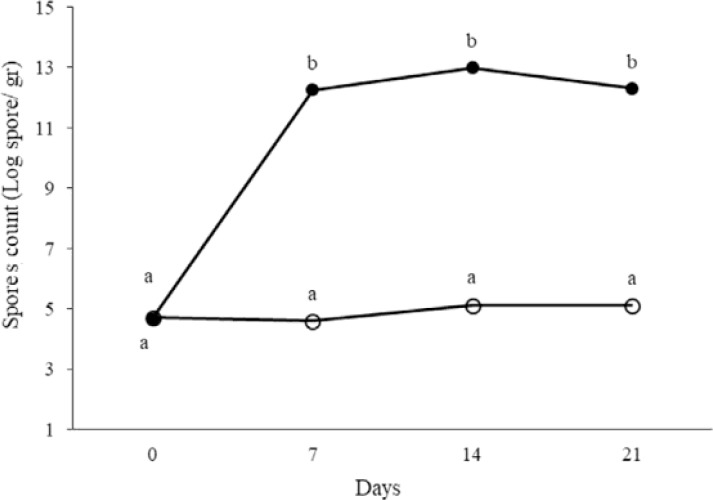
The logarithm of spore count in fecal samples of rat in control (○) and probiotic (●) groups. Different letters indicate significant differences between groups in each day (*p* < 0.05). Control: Received water for 3 weeks. Probiotic: Receiving water containing 5×10^7^ spores/ml of *B. subtilis* and 5×10^7^ spores/ml of *B. coagulans* for 3 weeks

At days 0, 7, 14 and 21 of treatment, total aerobic and anaerobic microorganisms, LAB, coliforms and spores in the fecal samples were counted. The results are shown in Figs. 1–5. Spore of *B. subtilis* and *B. coagulans* as probiotics, led to increase the total aerobic, LAB and spore counts significantly (*p* < 0.05). As the logarithm of total count of aerobic, LAB and spore in the last week were 9.76±0.83, 10.36±0.45 and 12.31±0.46 in order in probiotic group while they were respectively 8.47±0.42, 9.21±0.46, 5.11±0.23 in control group. Also, these probiotics caused decrease in anaerobe and coliform counts (*p* < 0.05). As the logarithms of total count of anaerobic and coliform were 8.06±0.33 and 5.1±0.17 in probiotic group while they were 8.76±0.35 and 6.28±0.33 in control group, respectively (*p* < 0.05).

## DISCUSSION

In the present study, addition of probiotics *B. subtilis* and *B. coagulans* in daily water caused significant enhancement of weight gain and decrease the feed intake. The FCR reduction which observed in this study suggests an improved intestinal balance of microbial population. Probiotics, improve the absorbtion of nutrients by stimulating the growth of beneficial bacteria and producing a healthier intestinal system (28, 29). Jager et al. showed that *B. coagulans* enhanced the absorption and utilization of proteins (24). In agreement with our findings, many studies showed that probiotics improve the FCR. For instance *B. subtilis* improve the FCR and average weight gain in the broiler chicken (25, 26). Also, *B. coagulans* improve the FCR in broiler chicken (19). Moreover, a mixture of *Lactobacillus, Bifidobacterium, Streptococcus, Enterococcus, Aspergillus* and *Candida* in broiler chicken increased the weight gain (30). *B. cereus* var. *toyoi* also increased average daily gain and body weight (31). Abdel Hamid et al. (2013) showed that *Lactobacillus spp*. enhanced the performance and decrease the mortality in contrast of antibiotics (32).

As mentioned before, spore of probiotics are resistance to gastric acid, so they will be able to pass through the stomach, and in a concentration similar to the initial concentration will enter the intestine (14). In the current study, *B. subtilis* and *B. coagulans* as probiotics, leaded to increase the total aerobic, lactic acid bacteria and spore counts and they decreased the anaerobe and coliform count. The *Bacillus* spores germinate in the gut (particularly in jejunum and ileum), proliferate, able to grow and resporulate (13, 33), and this cause the enhancement in aerobic and spore enumeration. The mechanisms by which probiotics exert biological effects are still poorly understood, but the nonspecific terms such as colonization resistance or competitive exclusion, antimicrobial substances, immunostimulatory effect, prevention of intestinal inflammation, and stimulation of growth of intestinal normal flora explain their mode of action (34, 35). Jin et al. (1996) showed that consumption of *B. subtilis* in feed as probiotic significantly increase the LAB and decrease the *E. coli* counts after 2 weeks of consumption and in 2011, Lin et al. resulted these changes for *B. coagulans* (19, 36). In contrast, another study showed that *B. subtilis* has no effect on the LAB and coliform numbers in turkey poults (37). While another study showed that *B. coagulans* and *B. pumilusi* are acid and bile tolerant, and as probiotics they reduced the fecal coliforms and enhanced the *Lactobacillus spp.* and spores of *Bacillus spp.* (17). LAB send signals to activate immune cells, so they induce good mucosal immunostimulation without inducing side effects such as bacterial translocation or a strong inflammatory immune response (38). Our findings showed that *B. subtilis* and *B. coagulans* as probiotic enhances LAB counts which is present in the intestinal tract of many animals and they have positive effect on protecting against intestinal pathophysiology (39). The intestinal microbiota limits the pathogens such as Salmonella infection, mechanism referred to as colonization resistance (40).

## CONCLUSION

*B. subtilis* and *B. coagulans* as probiotic cause beneficial effects on the intestinal microflora. As they increase the count of beneficial bacteria such as lactic acid bacteria and decrease the harmful ones such as coliforms, it can be recommended to use in food products as prevention way to reduce the foodborne disease.
